# The abscopal effect in metastatic lung cancer: a retrospective analysis of combined radiotherapy and immunotherapy

**DOI:** 10.1007/s10585-025-10375-w

**Published:** 2025-09-26

**Authors:** David Alexander Ziegler, Markus Ambold, Markus Anton Schirmer, Leif Hendrik Dröge, Sonia Ziegler, Benedikt Kieslich, Laura Anna Fischer, Sandra Donath, Jona Bensberg, Martin Leu, Manuel Guhlich, Lisa Antonia von Diest, Friederike Braulke, Alexander von Hammerstein-Equord, Stefan Andreas, Stefan Rieken, Achim Rittmeyer, Rami El Shafie

**Affiliations:** 1 Department of Radiotherapy and Radiation Oncology, University Medical Center Göttingen, Göttingen, Germany; 2 Department of Radiation Oncology and Radiotherapy, University Hospital Lausitz – Carl Thiem, Cottbus, Germany; 3 Department of Thoracic Oncology, Lungenfachklinik Immenhausen, Immenhausen, Germany; 4 Göttingen Comprehensive Cancer Center (G-CCC), University Medical Center Göttingen, Göttingen, Germany; 5 Department of Thoracic and Cardiac Surgery, University Medical Center Göttingen, Göttingen, Germany; 6 German Center for Lung Research, Giessen, Germany

**Keywords:** Abscopal effect, Lung cancer, Immunotherapy, NSCLC, SCLC

## Abstract

**Background:**

The abscopal effect (AbE) refers to the regression of non-irradiated tumors following localized radiotherapy (RT), suggesting a systemic immune-mediated response. The combination of RT with immunotherapy (IO) has been proposed to enhance the AbE by overcoming RT-induced immunosuppressive mechanisms, but robust evidence, specifically in non-small cell lung cancer (NSCLC), is lacking. This study aimed to determine the prevalence of the AbE and its association with progression-free survival (PFS) and overall survival (OS) in metastatic lung cancer patients receiving RT and IO.

**Materials and methods:**

We analyzed consecutive metastatic lung cancer patients who received IO at a certified lung cancer center in Germany between 2009 and 2021. Inclusion required RT to at least one lesion and at least one measurable non-irradiated lesion. The AbE was defined as radiological regression of a non-irradiated lesion following RT. PFS and OS were analyzed using Kaplan–Meier estimates and Cox regression models. Factors influencing survival, such as RT site selection and IO duration, were assessed in univariable and multivariable analyses.

**Results:**

A total of 72 patients were included. The AbE was observed in 9.3% (n = 7). While the AbE was significantly associated with prolonged PFS (HR = 0.41, 95% CI: 0.19–0.92, *p*  = 0.03), it did not significantly impact OS (HR = 0.70, 95% CI: 0.30–1.64, *p*  = 0.41). In contrast, RT to the primary tumor (HR = 0.16, *p* = 0.004) and longer IO duration (HR = 0.83, *p* = 0.001) were strong predictors of improved OS.

**Conclusion:**

While the AbE remains an intriguing phenomenon, its occurrence was rare and not significantly associated with OS. Strategic RT site selection and optimized treatment sequencing were more strongly linked to improved survival outcomes, highlighting the need for refined radioimmunotherapy strategies.

**Supplementary Information:**

The online version contains supplementary material available at 10.1007/s10585-025-10375-w.

## Introduction

The abscopal effect (AbE) describes tumor regression in non-irradiated lesions following localized radiotherapy (RT), suggesting a systemic immune-mediated response. First reports of the AbE date back to McCulloch in 1908 and Mole in 1953 [1, 2]. Ever since, the AbE remains a rare phenomenon, with most clinical evidence derived from case reports and small retrospective series, including heterogeneous tumor entities [3, 4]. The emergence of immunotherapy (IO) has renewed interest in the AbE, as preclinical studies indicate that RT-induced antigen release may enhance systemic anti-tumor immunity when combined with IO [5, 6].

Mechanistically, RT promotes immunogenic cell death, leading to increased tumor antigen presentation and dendritic cell activation [7]. However, RT also induces counter-regulatory mechanisms, including upregulation of programmed cell death 1 ligand 1 (PD-L1) and activation of transforming growth factor β (TGF-β), which can suppress anti-tumor immunity [8]. IO, by blocking these pathways, may enhance RT-induced immune responses, theoretically increasing the likelihood of an AbE [5, 9]. Clinical studies have reported enhanced response rates when RT is combined with IO in metastatic melanoma and non-small cell lung cancer (NSCLC), yet robust evidence demonstrating a survival benefit remains limited [10–13].

Recent studies in early-stage NSCLC further suggest that RT to the primary tumor may have unique immunomodulatory effects. The addition of stereotactic body radiotherapy (SBRT) to neoadjuvant durvalumab significantly increased major pathological response rates, including regression of non-irradiated lymph nodes, a form of localized AbE [14, 15]. These findings highlight the importance of RT site selection in generating systemic immune responses. However, in metastatic disease, the frequency of the AbE, its clinical impact, and the role of RT site selection remain poorly defined.

To address this, we conducted a retrospective analysis of metastatic lung cancer patients treated with combined RT and IO. Our primary objectives were to determine the prevalence of the AbE and its association with progression-free survival (PFS) and overall survival (OS), while assessing the impact of RT site selection and IO duration on clinical outcomes.

## Materials and methods

This retrospective study analyzed all consecutive patients diagnosed with metastatic lung cancer (UICC stage 4A/4B) and treated with IO, at a single certified lung cancer center in Germany over a 12-year period (2009–2021). The analysis specifically focused on patients who received additional concurrent RT to one or more symptomatic lesions. To assess the potential abscopal effect, inclusion criteria required patients to have at least one measurable non-irradiated lesion and to undergo cross-sectional imaging before and after RT (minimum of two pre-treatment scans).

Patients were excluded if they died within two weeks of completing RT or if their immunotherapy or systemic treatment was modified after RT. Patient data were retrieved from institutional databases and individual medical records.

### Patients and characteristics

The cohort included patients with the following lung cancer subtypes: small cell lung cancer (SCLC, n = 7, 9.7%), NSCLC adenocarcinoma (n = 45, 62.5%), NSCLC squamous cell carcinoma (n = 16, 22.2%), NSCLC not otherwise specified (NSCLC-NOS, n = 1, 1.4%), and large cell neuroendocrine carcinoma (LCNEC, n = 3, 4.2%). The majority of patients were male (n = 51, 70.8%), and the median age at RT initiation was 65 years. All patients had at least one metastasis. A history of smoking was reported in 86.1% (n = 62), while 5.6% (n = 4) were non-smokers; smoking history was unknown for 9.3% (n = 5). Additional patient characteristics are detailed in the results section.

### Radiotherapy (RT)

Seventy-two patients in this cohort underwent RT at various anatomical sites. Some received treatment at multiple sites simultaneously (n = 6/72, 8.3%), while others underwent multiple sequential RT courses (n = 10/72, 13.9%). RT doses ranged from 30 to 66 Gy, with an average total dose of 46.4 Gy and a median of 45 Gy. If a patient exhibited an AbE following multiple RT courses, only the session demonstrating this effect was included in the analysis. For patients without an AbE, the first RT session was designated as starting point T0 for PFS and OS estimation. Most patients received standard fractionated RT, while only two underwent stereotactic RT. Detailed RT regimens for patients receiving multiple sessions are provided in Supplementary Table 1.

### Immunotherapy (IO)

In this manuscript, we use the term IO to refer to agents such as anti-PD-1/PD-L1 antibodies. IO was required to start at least one day before RT and continue until RT completion. Modifications to the immunotherapy regimen were not permitted. The specific treatments administered are summarized in Table 1.

**Table 1:** Baseline parameters stratified for patients with versus without abscopal effect

	Total patient cohort n = 72 (100%)	Patients with abscopal effect n = 7 (100%)	Patients showing no abscopal effect n = 65 (100%)	Statistical testing
*Sex*				
Male	51 (70.8)	5 (71.4%)	48 (70.8%)	
Female	21 (29.2)	2 (28.6%)	19 (29.2%)	0.97 (χ^2^)
*Age*				
In years at radiotherapy (range)	65 (45–89)	58 (55–74)	65 (45–89)	0.10 (MWU)
*Smoking-status, n (%)*				
Current	18 (25.0)	3 (42.9)	15 (23.1)	
Former	44 (61.1)	4 (57.1)	40 (61.5)	
Never	4 (5.6)	0 (0)	4 (6.2)	
Unknown	6 (8.3)	0 (0)	6 (9.2)	0.56 (χ^2^)
> 10 py	57 (79.2)	7 (100)	50 (77)	0.36 (χ^2^)
*Histology, n (%)*				
SCLC	7 (9.7)	1 (14.3)	6 (9.2)	
AC	45 (62.5)	4 (57.1)	41 (63.1)	
SCC	16 (22.2)	1 (14.3)	15 (23.1)	
NOS	1 (1.4)	0 (0)	1 (1.5)	
LCNEC	3 (4.2)	1 (14.3)	2 (3.1)	0.66 (χ^2^)
*Immunotherapy, n (%)*				
Atezolizumab	22 (30.6)	3 (42.9)	19 (29.2)	
Nivolumab	19 (26.4)	4 (57.1)	15 (23.1)	
Pembrolizumab	29 (40.3)	0 (0)	29 (44.6)	
Nivo-/Iplimumab	1 (1.4)	0 (0)	1 (1.5)	
Durvalumab	1 (1.4)	0 (0)	1 (1.5)	0.17 (χ^2^)
*Radiotherapy target, n (%)*				
Primary tumor	17 (23.6)	1 (14.3)	16 (24.6)	
Pleura	3 (4.2)	0 (0)	3 (4.6)	
Cerebrum	17 (23.6)	3 (42.9)	14 (21.5)	
Adrenal gland	6 (8.3)	1 (14.3)	5 (7.7)	
Extrathoracic lymp hnodes	3 (4.2)	0 (0)	3 (4.6)	
Bone	20 (27.8)	2 (28.6)	18 (27.7)	
Connective tissue	1 (1.4)	0 (0)	1 (1.5)	
Two sites *	5 (6.9)	0 (0)	5 (7.7)	0.87 (χ^2^)

* = Two sites irradiated: 1 × bone + primary tumor, 1 × bone + cerebrum, 1 × bone + adrenal gland, 1 × bone + mediastinum, 1 × extrathoracic lymph nodes + connective tissue). SCLC, Small cell lung cancer; AC, NSCLC adenocarcinoma; SCC, NSCLC squamous cell carcinoma; NOS, not otherwise specified carcinoma; LCNEC, large cell neuroendocrine carcinoma; χ^2^, Chi-squared test

### Definition of abscopal effect

For this study, the AbE was identified as a response in a lesion outside the RT target area (receiving less than 10% of the prescribed radiotherapy dose). Medical imaging techniques, such as CT or PET-CT, were used to detect AbEs, which were then confirmed by a lung cancer specialist. The evaluation was conducted using the iRECIST criteria [16].

### Statistical analysis

Baseline data are presented descriptively using means and standard deviations (SD) for continuous variables, and medians with quartiles and ranges where appropriate. Categorical variables are reported as absolute and relative frequencies. OS was defined as the time from first day of first radiation session to death or the last recorded observation. PFS was defined as the time from first irradiation to tumor progression, death, or the last recorded observation.

Univariable and multivariable Cox regression analyses were performed to identify prognostic factors for PFS and OS. Variables with a p-value < 0.2 in the univariable analysis and meeting statistical criteria for multivariable modeling (subgroup size and event count) were included in the multivariable Cox regression. Among highly correlated variables, the most clinically relevant was selected for inclusion. Given the retrospective design, statistical associations are considered exploratory, with statistical significance defined as p < 0.05.

All statistical analysis were conducted using IBM SPSS Statistics, Version 22 (New York, USA). The study was approved by the institutional ethical review board (approval number: 38/4/22, May 9th 2022).

## Results

**Fig. 1:**
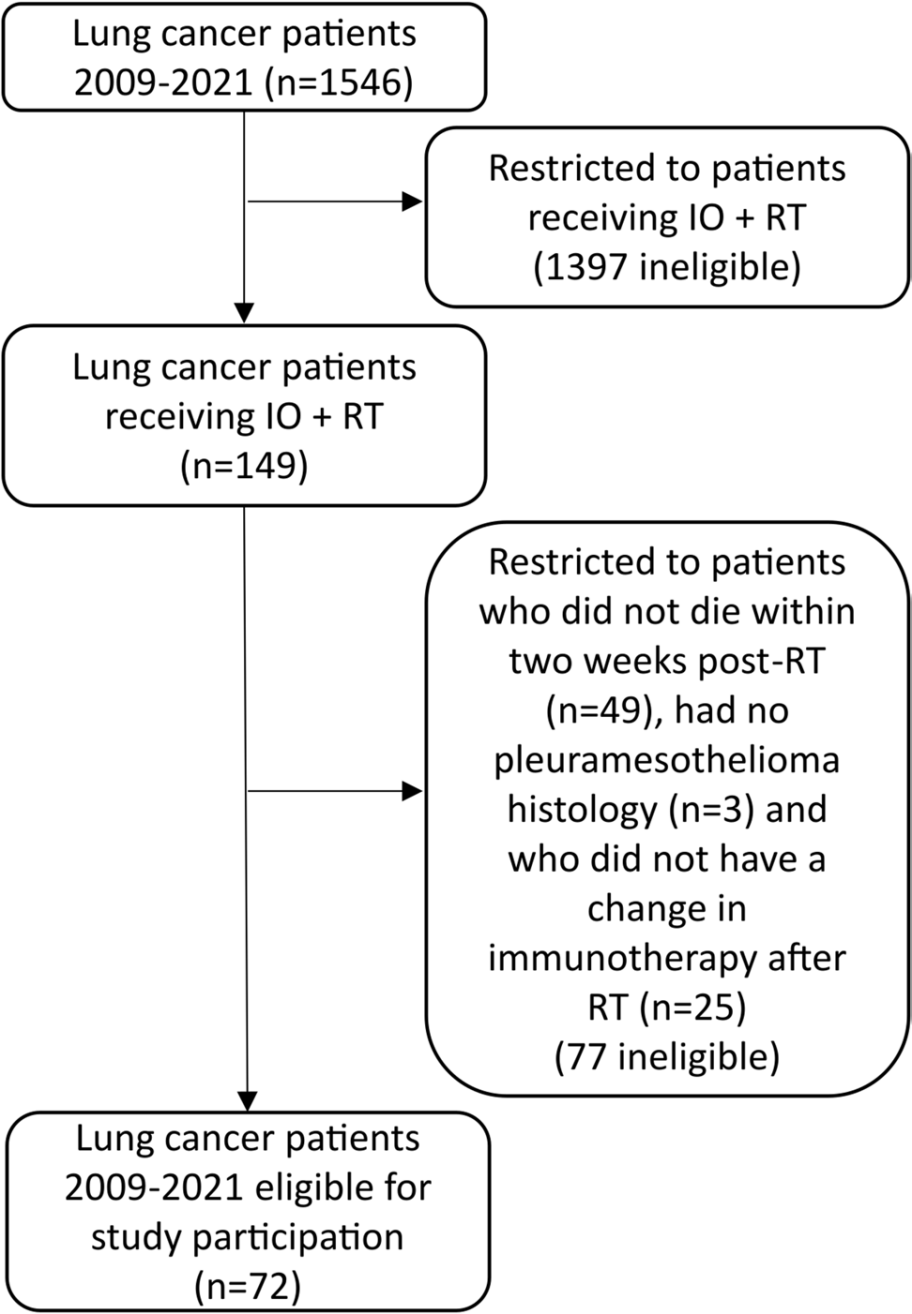
Flow diagram patients enrollment IO, immunotherapy; RT, radiotherapy

From an initial cohort of 1,546 lung cancer patients treated between 2009 and 2021, 149 received concurrent radioimmunotherapy (see Flowchart, Fig. 1). After excluding those who died within two weeks post-RT (n = 49) or had changes in immunotherapy after RT (n = 25), 72 patients remained in the final analysis. The cohort had a median age of 65 years, with 70.8% being male. The most common histological subtypes were NSCLC adenocarcinoma (62.5%) and squamous cell carcinoma (22.2%), while 9.7% had small cell lung cancer (SCLC). Immunotherapy regimens included pembrolizumab (40.3%), atezolizumab (30.6%), and nivolumab (26.4%). Radiotherapy was most frequently administered to the primary tumor (23.6%), bone metastases (34.8%), or cerebral metastases (25%). Further details on patient characteristics are provided in Table 1.

### Overall survival (OS)

**Fig. 2:**
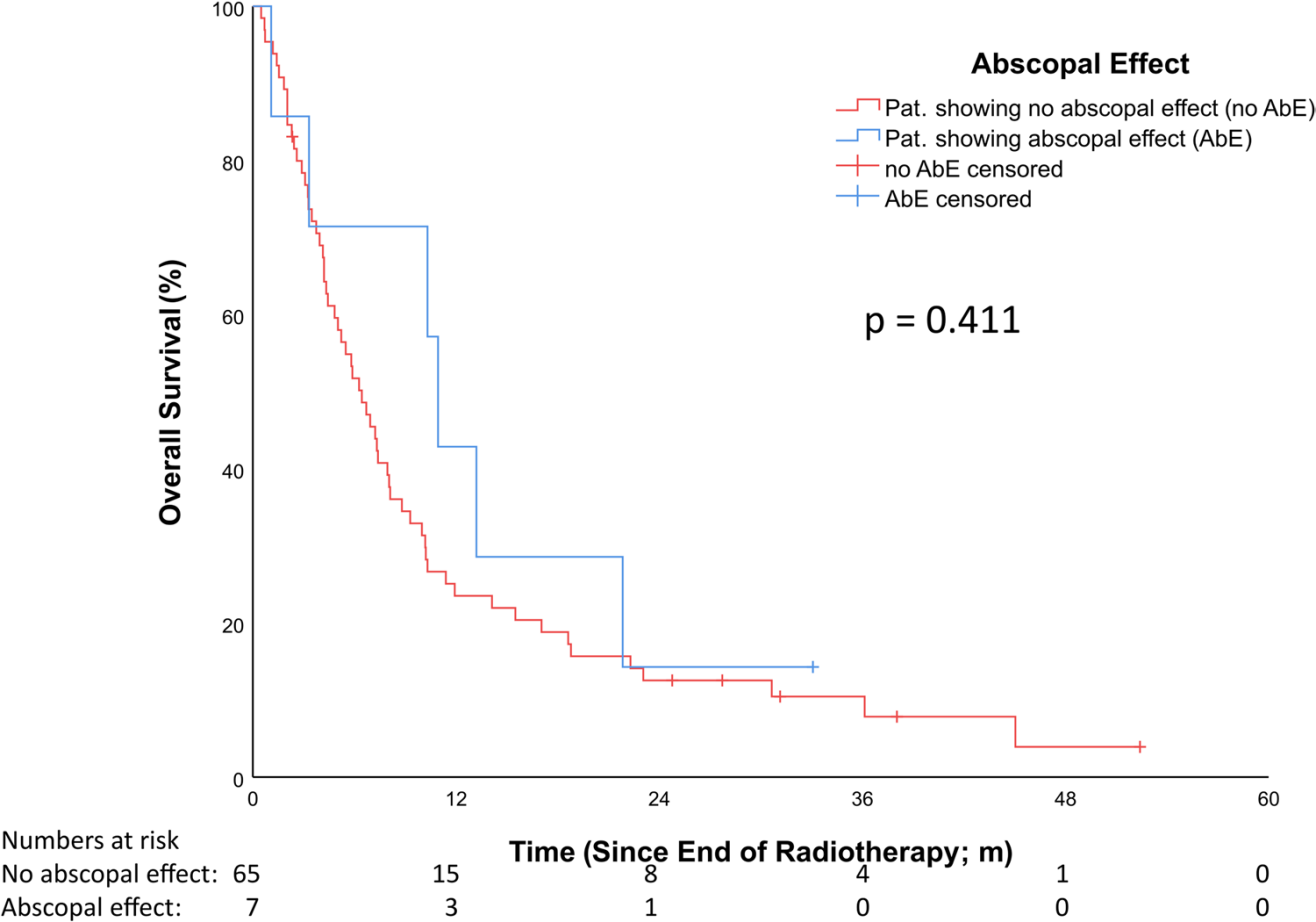
Results overall survival; *p* = 0.411

The median follow-up for OS, estimated using the reverse Kaplan–Meier method, was 39.4 months (Q1–Q3: 32.7–52.8; 95% CI: 31.52–47.27). Median OS was 7.1 months (Q1–Q3: 4.0–13.3; 95% CI: 5.1–9.0) in patients without an AbE and 12.3 months (Q1–Q3: 4.3–22.6; 95% CI: 9.5–10.0) in patients with an AbE (HR = 0.7, 95% CI: 0.3–1.64, p = 0.41). OS at 12 months was 23.1% (n = 15/65) in the non-AbE group and 57.1% (n = 4/7) in the AbE group (see Fig. 2).

Univariable Cox regression analysis identified several significant associations with OS. RT to the primary tumor was significantly associated with improved OS (HR = 0.48, 95% CI: 0.26–0.90, *p* = 0.021). In contrast, RT to the cerebrum was associated with an increased risk of mortality (HR = 2.37, 95% CI: 1.32–4.25, *p* = 0.004). A longer interval from RT initiation to the end of IO therapy correlated with improved OS (HR = 0.86, 95% CI: 0.81–0.92, *p* < 0.001). TP53 mutations showed a trend towards worse OS (HR = 1.92, 95% CI: 0.92–4.01, *p*=  0.08). The presence of an AbE was not significantly associated with OS (HR = 0.7, 95% CI: 0.3–1.64, *p* = 0.41). Other variables—including sex, histology, PD-L1 expression, radiation dose, age, and immunotherapy—did not reach statistical significance (see Table 2).

**Table 2:**
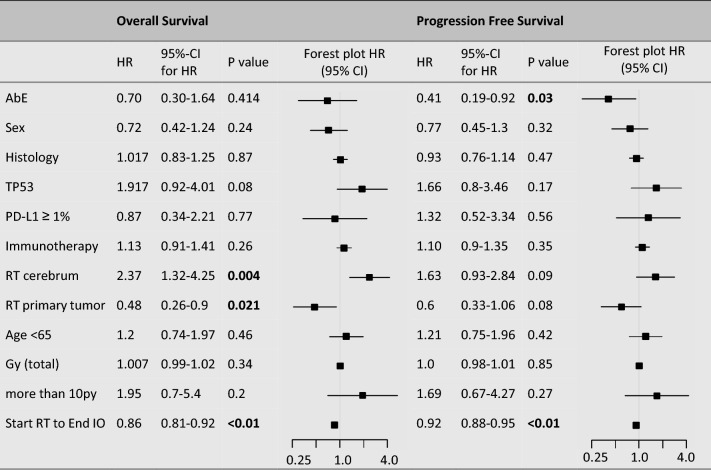
Univariable COX-analysis

AbE, abscopal effect; Sex, male/female; Histology, tumor histology type; TP53, tumor protein p53 mutation status (mutated/non-mutated); PD-L1 ≥1%, programmed death-ligand 1 expression (immunohistochemistry score); Immunotherapy, type of immune checkpoint inhibitor used; RT cerebrum, radiotherapy to the cerebrum versus other sites; RT primary tumor, radiotherapy to the primary tumor versus other locations; Age  < 65, patients younger than 65 years at treatment initiation; Gy (total), total administered radiation dose in Gray; more than 10 py, smoking history ( > 10 pack-years); Start RT to End IO, time from RT initiation to the end of immunotherapy (days). Bold values indicate statistical significance (*p*  < 0.05)

In multivariable Cox regression analysis, the time from RT initiation to the end of IO remained a significant predictor of OS (HR = 0.83, 95% CI: 0.74–0.93, *p*  = 0.001). RT to the primary tumor remained significantly associated with OS (HR = 0.16, 95% CI: 0.05–0.55, *p* = 0.004). The AbE did not reach statistical significance in the multivariable model (HR = 1.53, 95% CI: 0.4–5.9, *p* = 0.54). RT to the cerebrum also did not show a significant association with OS (HR = 0.53, 95% CI: 0.19–1.5, *p* = 0.23). For detailed numerical results, see Table 3.

**Table 3:**
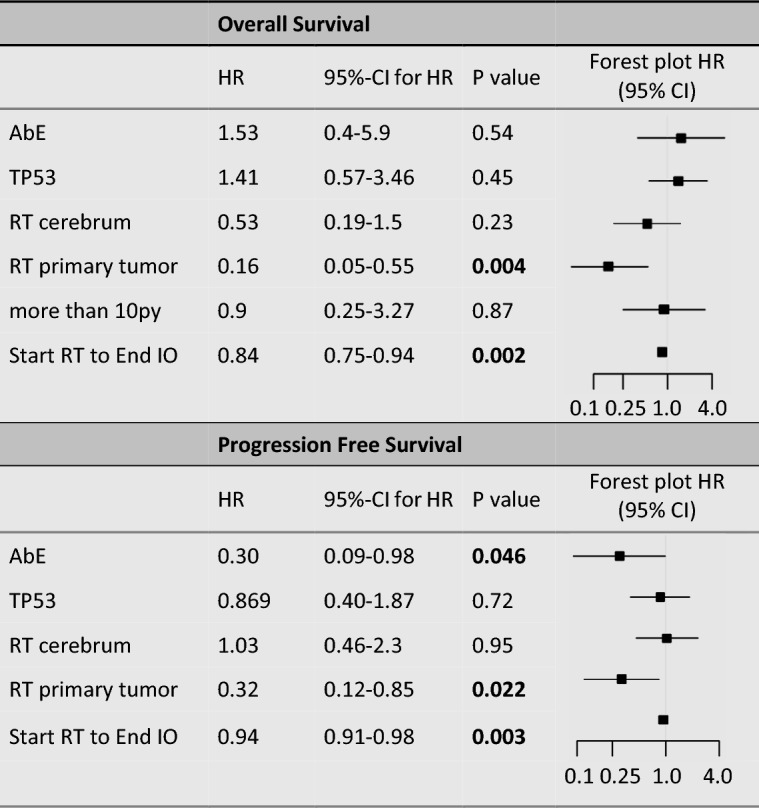
Multivariable COX-analysis (including variables of univariat COX-analysis with *p* value  <0.1)

AbE, abscopal effect; TP53, tumor protein p53 mutation status (mutated/non-mutated); RT cerebrum, radiotherapy to the cerebrum versus other sites; RT primary tumor, radiotherapy to the primary tumor versus other locations; more than 10 py, smoking history ( >10 pack-years); Start RT to End IO, time from RT initiation to the end of immunotherapy (days). Bold values indicate statistical significance (*p*  < 0.05)

### Progression free survival (PFS)

**Fig. 3:**
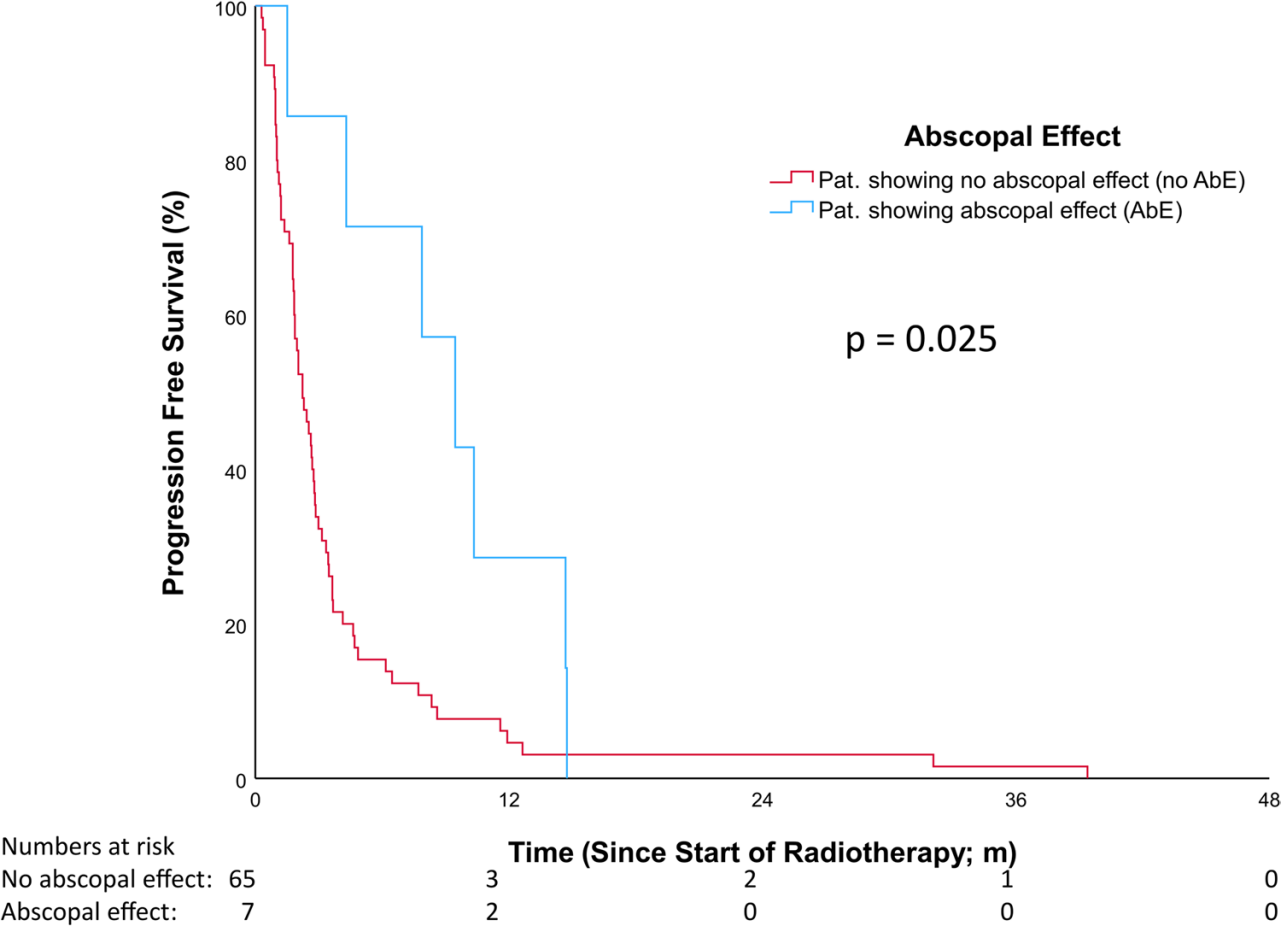
Results progression free survival; *p* = 0.025

The median PFS, evaluated using the Kaplan–Meier method, was significantly longer for patients exhibiting an AbE (9.46 months, Q1–Q3: 4.3–14.7; 95% CI: 5.42–13.51) compared to those without an AbE (2.23 months, Q1–Q3: 1.21–3.65; 95% CI: 1.6–2.87) (HR = 0.41, 95% CI: 0.19–0.92, *p* = 0.03). At 12 months, 28.6% (n = 2/7) of AbE patients remained progression-free compared to 4.6% (n = 3/65) in the non-AbE group (see Fig. 3).

Univariable Cox regression analysis identified several significant associations with PFS. The presence of an AbE was significantly correlated with improved PFS (HR = 0.41, 95% CI: 0.19–0.92, *p*  = 0.03). A longer interval from RT initiation to the end of IO was also strongly associated with prolonged PFS (HR = 0.92, 95% CI: 0.88–0.95, *p* < 0.01). RT to the cerebrum showed a trend toward worse PFS (HR = 1.63, 95% CI: 0.93–2.84, *p* = 0.09), while RT to the primary tumor site showed a near-significant trend toward improved PFS (HR = 0.6, 95% CI: 0.33–1.06, *p*  = 0.08). Other variables—including sex, histology, PD-L1 expression, immunotherapy, TP53 mutations, age at RT initiation, and total radiation dose—were not significantly associated with PFS (see Table 2 for details).

In multivariable Cox regression analysis, the time from RT initiation to the end of IO remained a significant predictor of prolonged PFS (HR = 0.94, 95% CI: 0.91–0.98, *p* = 0.003). RT to the primary tumor site was also significantly associated with PFS (HR = 0.32, 95% CI: 0.12–0.85, *p* = 0.022). Additionally, the AbE retained statistical significance in the multivariable model (HR = 0.30, 95% CI: 0.09–0.98, *p* = 0.046). RT to the cerebrum and TP53 mutations did not show significant associations with PFS (see Table 3 for details).

### Detailed cases of observed AbEs

Table 4 provides an overview of patients who experienced an AbE. The duration of IO, RT, and changes in the size of the abscopal organ are detailed in the following subsection. Among these patients, the median dose per fraction was also 3 Gy (range 1.8–3 Gy), reflecting the dosing pattern of the entire cohort, where the median dose per fraction was likewise 3 Gy (range 1.8–5 Gy). One illustrative case is described below, while the remaining cases can be found in the supplementary material (see Supplementary Cases 1–6).

**Table 4:** Patients with abscopal effects after radioimmunotherapy

Pat	4	7	8	9	10	14	16
Age In years at radiotherapy	55	58	74	57	71	61	56
Sex (m / f)	m	f	f	m	m	m	m
Histology	LCNEC	AC	AC	SCLC	AC	SCC	AC
Immunotherapy	Nivolumab	Nivolumab	Atezolizumab	Atezolizumab	Atezolizumab	Nivolumab	Nivolumab
RT region	Osseus metastasis	Adrenal gland	Cerebrum	Primary tumor	Osseus metastasis	Cerebrum	Cerebrum
Total dose delivered (Gy)	30	45	30	50.4	39	40	45
Dose per fraction (Gy)	3	3	3	1.8	3	2.5	3
Abscopal location	Primary tumor + Adrenal gland	Primary tumor + Cerebrum	Primary tumor	Cerebrum	Primary tumor	Primary tumor	Primary tumor
AbE time RT to AbE in days	113	47	27	51	33	55	136
Time AbE to no AbE in days	313	246	6	149	386	46	126
Time begin IO to AbE	301	256	418	378	1270	208	209

Age, patient age in years at the time of radiotherapy; Sex, male (m) or female (f); Histology, tumor histology type (LCNEC, large cell neuroendocrine carcinoma; AC, adenocarcinoma; SCLC, small cell lung cancer; SCC, squamous cell carcinoma); Immunotherapy, type of immune checkpoint inhibitor administered; RT region, primary site of radiotherapy; Abscopal location, site where the abscopal effect (AbE) was observed; AbE time RT to AbE in days, time from radiotherapy initiation to the occurrence of the abscopal effect (days); Time AbE to no AbE in days, duration of the abscopal effect before resolution (days); Time begin IO to AbE, time from the start of immunotherapy to the occurrence of the abscopal effect (days)

**Fig. 4:**
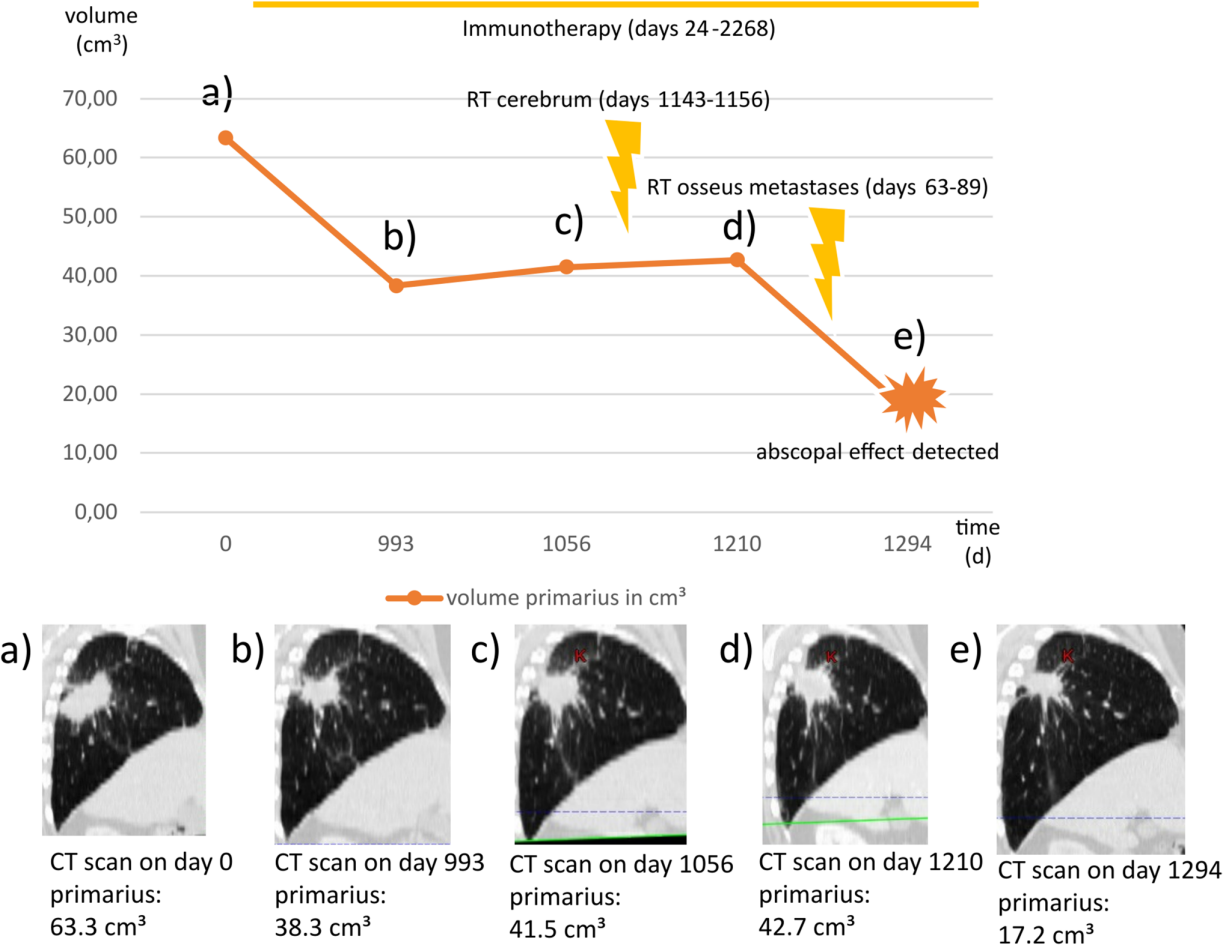
Patient 10

Patient 10 (Fig. 4) received IO for approximately 2244 days and underwent radiation therapy to the cerebrum from day 1143 to day 1156 after the baseline CT for IO. Radiation therapy of an osseous metastasis was performed from day 1233 to day 1261 after the baseline CT for IO. The AbE was noted in the primary tumor. Initially, the tumor volume was 63.3 cm^3^ on day 0, decreasing to 38.3 cm^3^ by day 993, then slightly increasing to 41.5 cm^3^ by day 1056 and 42.7 cm^3^ by day 1210, before finally reducing to 17.2 cm^3^ by day 1294. This reduction from 42.7 cm^3^ to 17.2 cm^3^ was a 59.7% decrease. The AbE was shown 84 days after the completion of the last radiation therapy. The corresponding graphical evidence is shown below.

## Discussion

This study demonstrates that the abscopal effect occurs in roughly every tenth patient with metastatic lung cancer patients simultaneously undergoing radiotherapy and immunotherapy and is significantly associated with prolonged PFS. However, in our study the AbE was not significantly associated with OS. In contrast, RT to the primary tumor and longer IO duration were strongly associated with improved OS. These findings suggest that while the AbE may contribute to tumor control, other factors, such as RT site selection and treatment duration, may play a more decisive role in long-term survival.

### The abscopal effect and PFS

The primary observation of our study was that the occurrence of an AbE was associated with a significant improvement in PFS, suggesting that the immune-mediated systemic response elicited by RT contributes to delaying disease progression. This is consistent with preclinical studies that have demonstrated RT-induced immunogenic cell death, leading to increased tumor neoantigen presentation and enhanced cytotoxic T cell responses [5, 6].

Several mechanistic factors might explain why the AbE is more closely linked to PFS rather than OS. First, the immune-modulating effects of RT may initially enhance anti-tumor immunity, leading to delayed disease progression. However, subsequent tumor adaptations, such as immune escape mechanisms, may eventually limit long-term survival benefits [8, 17]. Additionally, given the highly heterogeneous nature of metastatic lung cancer, other disease-related factors, including tumor burden and microenvironmental resistance, could overshadow the survival benefits of an AbE.

### The abscopal effect and OS

While the AbE was associated with prolonged PFS, it did not significantly impact OS. However, given the small patient cohort and the direction of the hazard ratio (HR = 0.70), a potential positive effect on OS cannot be ruled out. In contrast, RT to the primary tumor and longer IO duration were both strong and statistically significant predictors of improved OS.

One likely explanation is that RT to the primary tumor may exert broader immunomodulatory effects than RT to metastatic sites. Prior studies have suggested that lymphoid-rich regions, such as the mediastinum, play a key role in systemic immune activation [18, 19]. In our cohort, patients receiving RT to the primary tumor had significantly improved OS, possibly due to enhanced antigen presentation, T-cell priming, or modulation of lymphatic drainage pathways. These findings align with results from the PEMBRO-RT and MDACC trials, which demonstrated that RT combined with pembrolizumab improved abscopal responses, disease control, and OS in metastatic NSCLC patients [18, 19]. However, it is important to note that those trials assessed more homogeneous cohorts (exclusively NSCLC, pembrolizumab, and pulmonary metastases).

Another key factor influencing OS in our study was the duration of IO following RT. Patients who received prolonged IO had significantly longer OS, consistent with prior studies showing that continuous checkpoint inhibition can sustain anti-tumor immunity even after initial progression [5, 9]. However, in our cohort, IO was not modified at the time of RT, and only a single progressive lesion was irradiated, regardless of other disease progression. This may have limited the potential benefit of RT, as patients had already developed systemic resistance mechanisms before irradiation was administered. Furthermore, our inclusion criteria required patients to have progressive disease under IO, making our cohort inherently more resistant to immune therapy. These factors likely contributed to the shorter overall PFS in our study compared to trials with earlier-line IO and RT integration.

### Mechanistic considerations

The molecular mechanisms underlying the AbE remain an area of active investigation.

A notable clinical example was reported by Postow et al., who observed a pronounced abscopal response in a patient with metastatic melanoma treated with ipilimumab and localized radiotherapy. This effect was accompanied by increased NY-ESO-1–specific antibody levels, enhanced CD4⁺ T-cell activation, and reduced levels of myeloid-derived suppressor cells, indicating systemic immune activation triggered by local RT [20].

Most previously reported abscopal effects have been observed following hypofractionated or stereotactic radiotherapy, typically using doses of 6 to 8 Gy per fraction [7, 21]. However, several case reports and small series have also documented abscopal responses after conventionally fractionated RT, indicating that such effects may not be strictly limited to high-dose regimens, though they appear to be less frequent [3].

RT is known to enhance antigen presentation by upregulating major histocompatibility complex (MHC) class I expression, thereby increasing tumor visibility to cytotoxic T cells [22]. Additionally, RT induces the release of damage-associated molecular patterns (DAMPs), which activate dendritic cells (DCs) and cross-prime tumor-specific T cells [7]. These processes are essential for mounting a systemic anti-tumor immune response and likely play a role in the AbE.

However, our findings suggest that not all RT sites exert the same immunologic impact. While the AbE was associated with prolonged PFS, it did not improve OS, whereas RT to the primary tumor was a strong predictor of OS (HR = 0.16, *p* = 0.004). This distinction aligns with findings from neoadjuvant trials in early-stage NSCLC investigating SBRT combined with immunotherapy. In a randomized phase 2 trial, Altorki et al. demonstrated that adding SBRT to neoadjuvant durvalumab significantly increased major pathological response (MPR) rates from 6.7% to 53.3% (*p* < 0.0001), suggesting that RT to the primary tumor can act as a potent immune sensitizer​ [14]. Similarly, a recent phase 2 study by Zhao et al. found that SBRT followed by sequential tislelizumab and chemotherapy led to an MPR rate of 76% and a pCR rate of 52%, far exceeding historical controls ​[15]. Crucially, both studies reported a form of the AbE – lymph node clearance – where non-irradiated metastatic LNs regressed following RT to the primary tumor.

This phenomenon suggests that the immunomodulatory effects of RT are most pronounced in tumor-draining lymph nodes (TDLNs), where antigen presentation and T cell priming occur. Preclinical and clinical studies indicate that lymphoid-rich environments facilitate stronger systemic immune responses compared to isolated distant metastases [18, 19]. This could contribute to our finding that RT to the primary tumor – but not the AbE in distant metastases – was associated with OS improvement.

Additionally, while RT can trigger an initial immune response, it also induces immunosuppressive pathways, including PD-L1 upregulation and TGF-β activation, which can blunt long-term efficacy [8]. These findings reinforce the idea that treatment sequencing, site selection, and immune resistance mechanisms must be considered when optimizing radioimmunotherapy approaches [9].

### Clinical implications and future directions

Our findings reinforce the growing evidence that the interaction between RT and IO is highly dependent on treatment site selection, sequencing, and immune resistance mechanisms. While the AbE remains an intriguing phenomenon, our data suggests that it is relatively rare and may not be the most reliable predictor of survival outcomes. Instead, RT to the primary tumor and prolonged IO duration were stronger predictors of OS, highlighting the importance of treatment strategies that prioritize immune priming in lymphoid-rich environments. This aligns with findings from early-stage NSCLC trials, where SBRT to the primary tumor significantly enhanced pathological response rates and lymph node clearance in the neoadjuvant setting​ [14, 15].

Based on our findings, several preliminary recommendations can be made for current clinical practice. Radiotherapy to the primary tumor – particularly in lymphoid-rich regions such as the mediastinum – was associated with improved overall survival and may be preferred over irradiation of distant metastases when systemic immune modulation is the intended goal. Previous reviews have reported that abscopal effects are more frequently observed following hypofractionated or stereotactic radiotherapy, likely due to enhanced immunogenic cell death and antigen presentation associated with higher dose per fraction [3, 23]. While most of these cases involved doses of 6–8 Gy per fraction, our data suggest that conventional fractionation (e.g., 2–3 Gy per fraction) may also elicit systemic responses in selected cases, particularly when delivered without significant delays. In the absence of predictive biomarkers, RT should ideally be timed during ongoing checkpoint inhibition. Even within current clinical frameworks, thoughtful site and timing selection may influence systemic immune effects.

These insights have important clinical implications. The results suggest that RT to the primary tumor should be prioritized over palliative RT to metastases when the goal is systemic immune modulation. Future trials should explore how to maximize the immune-boosting effects of RT through strategic site selection and combination approaches targeting immunosuppressive pathways such as PD-L1 and TGF-β. In this context, recent studies have explored whether the abscopal effects of radiotherapy can be amplified by targeting immune evasion pathways such as CD47. CD47 serves as a "don't eat me" signal, enabling tumor cells to escape phagocytosis by macrophages. Blocking this pathway may increase the immunogenicity of irradiated tumor cells and facilitate systemic antitumor responses. A study by Wang et al. [24] investigates the potential of combining radiotherapy with CD47 blockade to boost immune-mediated tumor control. Similarly, Nishiga et al. [25] demonstrated in a preclinical model that the combination of radiotherapy and anti-CD47 therapy elicited robust abscopal responses, even in the absence of T cells, highlighting a critical role for macrophages in mediating this effect. The promising results from preclinical studies with bintrafusp alfa, a dual PD-L1/TGF-β inhibitor, warrant further investigation in this context [8]. Additionally, recent data indicate that radiation fractionation schedules and sequencing with IO may significantly impact immune priming, underscoring the need for prospective trials assessing optimal dose, fractionation, and timing. Several ongoing studies are working toward defining these parameters. The yDEGRO multicenter study [26] aims to refine our understanding of the conditions under which the AbE is most likely to occur. Additionally, biomarker discovery remains an urgent priority—recent studies suggest that circulating tumor DNA (ctDNA) dynamics, T cell receptor (TCR) repertoire expansion, and immune-related gene expression signatures may serve as indicators of effective systemic immune activation following RT [27–29]. Identifying such biomarkers could help stratify patients most likely to benefit from combined RT and IO approaches, paving the way for more personalized radioimmunotherapy strategies.

### Strengths and limitations

Our study is strengthened by the valuable insights it provides into the interaction between RT and IO in metastatic lung cancer and is one of the largest retrospective analyses specifically assessing the AbE in this setting. A key strength is the detailed evaluation of treatment sequencing, RT site selection, and their impact on long-term survival outcomes. Unlike previous case reports and small series, our study provides real-world evidence that the AbE, while measurable, may be less clinically relevant than strategic RT site selection and IO duration.

Several limitations of our study must be acknowledged. First, as a retrospective single center study, causal conclusions cannot be drawn, and prospective validation in randomized trials is needed. Second, the relatively small number of AbE-positive patients (9.3%) limits the statistical power to detect subtle survival differences. Third, the study population was heterogeneous in terms of histology, RT dose, and IO regimen, which may introduce variability in treatment responses. Future studies should focus on more homogeneous cohorts with standardized RT and IO protocols.

A further limitation of our study is the lack of immunologic correlates such as T-cell receptor repertoire dynamics or serum-based biomarkers. As this was a retrospective analysis of patients treated between 2009 and 2021, paired biospecimens before and after radiotherapy were not available. Consequently, we could not assess systemic immune activation directly, and the classification of abscopal responses relied solely on radiographic criteria.

Another limitation of this study is the lack of systematic toxicity data. As RT was administered at different external centers, we were unable to collect standardized information on acute or chronic side effects. However, no patient required a treatment interruption longer than three days, suggesting overall good tolerability. Given the average total dose of 45 Gy and a median dose per fraction of 3 Gy, significant toxicities were not expected. Nonetheless, the impact of treatment-related toxicity on clinical outcomes such as the abscopal effect or survival could not be formally assessed.

## Conclusion

In conclusion, our study demonstrates that while the AbE is a real phenomenon, it remains rare and was not a significant predictor of OS. Instead, RT to the primary tumor and prolonged IO duration were the strongest survival determinants, emphasizing the importance of treatment site selection and therapy sequencing in optimizing radioimmunotherapy outcomes.

**Fig. 5:**
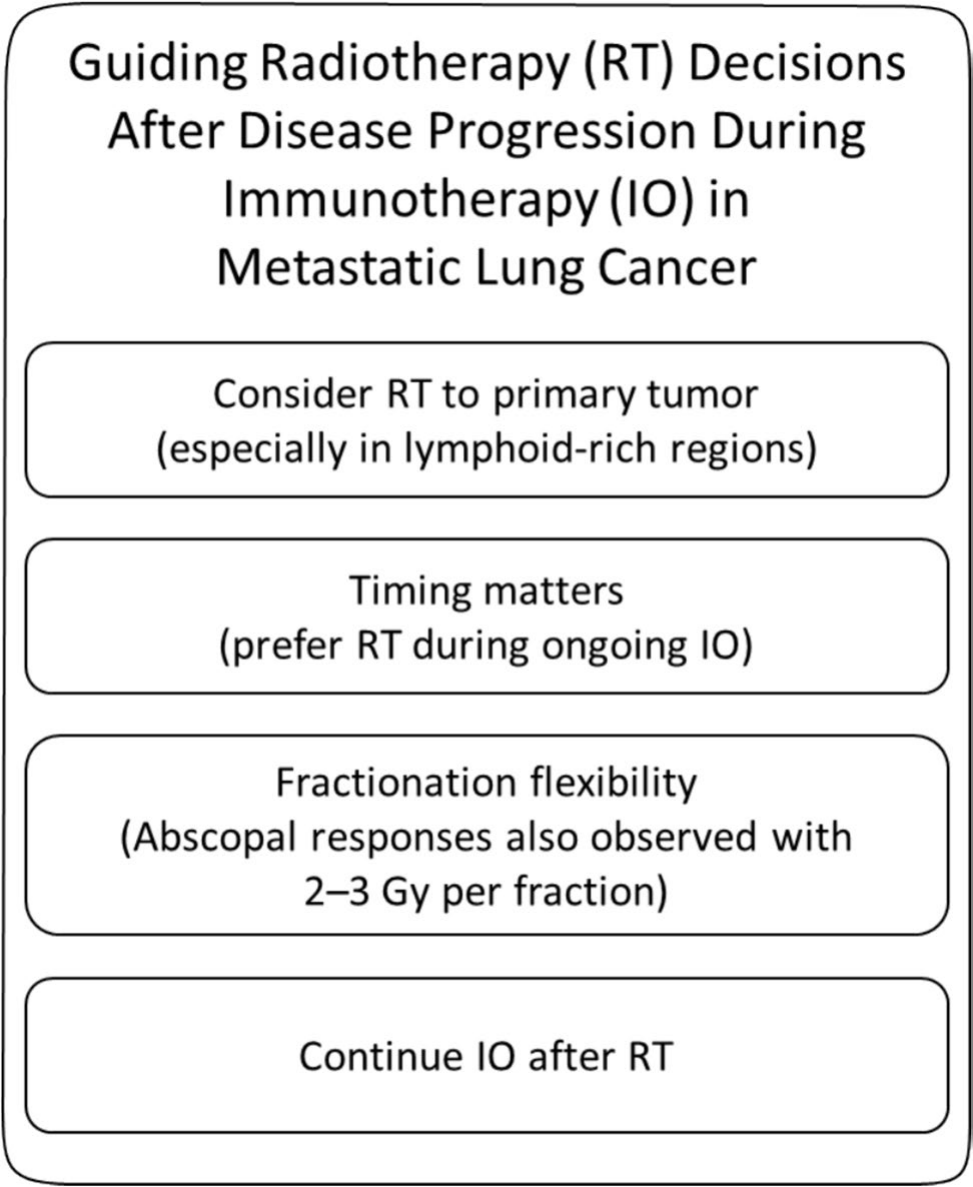
Structured summary of key clinical takeaways for radiotherapy during immunotherapy in metastatic lung cancer

Our findings support the preferential use of radiotherapy to the primary tumor—particularly in lymphoid-rich areas—over distant metastases when systemic immunomodulation is desired. Additionally, concurrent timing of RT during ongoing checkpoint inhibition and prolonged immunotherapy duration appears beneficial. These insights may already guide current clinical decision-making, even as further research continues to refine dose, fractionation, and biomarker selection to optimize systemic immune activation in metastatic lung cancer. A summary of these clinical recommendations is provided in Fig. 5.

## Supplementary Information

Below is the link to the electronic supplementary material.

Supplementary file1 (DOCX 1311 KB)

## Data Availability

Due to the inclusion of patient-related information and medical imaging data, the dataset is not publicly available but can be provided by the corresponding author upon reasonable request.
