# Supplementary material for: The abscopal effect in metastatic lung cancer: a retrospective analysis of combined radiotherapy and immunotherapy
Source: Clin Exp Metastasis. 2025 Sep 26;42(5):56. doi: 10.1007/s10585-025-10375-w (PMC12474601; doi:10.1007/s10585-025-10375-w)
Supplement: Supplementary file 1 — Supplementary file1 (DOCX 1311 KB) [file 10585_2025_10375_MOESM1_ESM.docx]

**Supplementary material**

Suppl Table 1. Patients receiving multiple courses of Radiotherapy

| Pat. | 9 | 10 | 19 | 22 | 31 | 45 | 60 | 64 | 66 | 101 |
| --- | --- | --- | --- | --- | --- | --- | --- | --- | --- | --- |
| Age  In years at radiotherapy | 57 | 71 | 60 | 62 | 45 | 48 | 60 | 58 | 71 | 66 |
| Sex (m / f) | m | m | m | m | m | m | w | w | w | m |
| Histology | SCLC | AC | AC | AC | AC | Pl | AC | AC | AC | AC |
| Immunotherapy | Ate | Ate | Pb | NI | Niv | Niv | Pb | Pb | Pb | Pb |
| RT region 1 RT region 2 RT region 3 | PT PT | Cer Oss | Oss AG | Oss Oss | AG PT | Ple Ms PT | PT Oss | PT Cer | AG PT | PT Oss |
| Abscopal effect | yes | yes | yes | no | no | no | no | no | no | no |
| m, male; f, female; SCLC, small cell lung cancer; AC, NSCLC adenocarcinoma; Pl, Pleuramesothelioma; Ate, Atezolizumab; Pb, Pembrolizumab; NI, Nivolumab-Imiplumab; Niv, Nivolumab; PT, primary tumor; Cer, cerebrum; Oss, osseous metastasis; AG, adrenal gland; Ple, pleural; Ms, muscle; Cer, cerebrum | | | | | | | | | | |


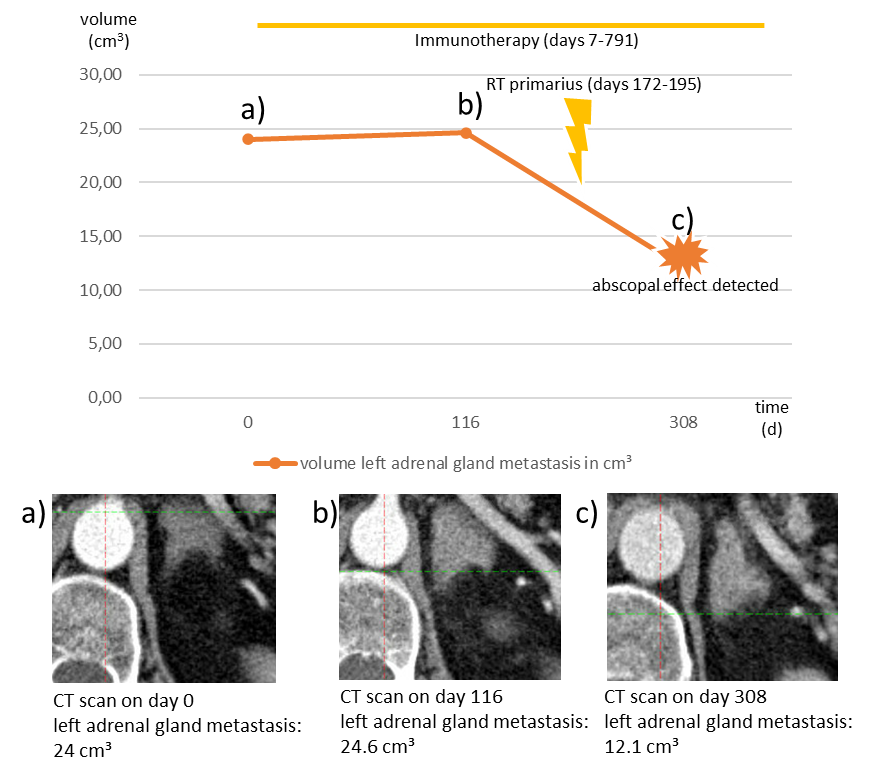
Suppl Case 1:

Patient 4 (Suppl Fig. 1) received IO for approximately 785 days and underwent radiation therapy to an osseous metastasis from day 172 to day 195 after the baseline CT for IO. The AbE was observed in the left adrenal gland metastasis. The volume of the metastasis was 24 cm³ on day 0, increased to 24.6 cm³ by day 116, and then decreased to 12.1 cm³ by day 308. This reduction from 24.6 cm³ to 12.1 cm³ was a 50.8% decrease. The AbE was shown 192 days after the completion of radiation therapy. The corresponding graphical evidence is shown below.

Suppl Fig. 1 - Patient 4


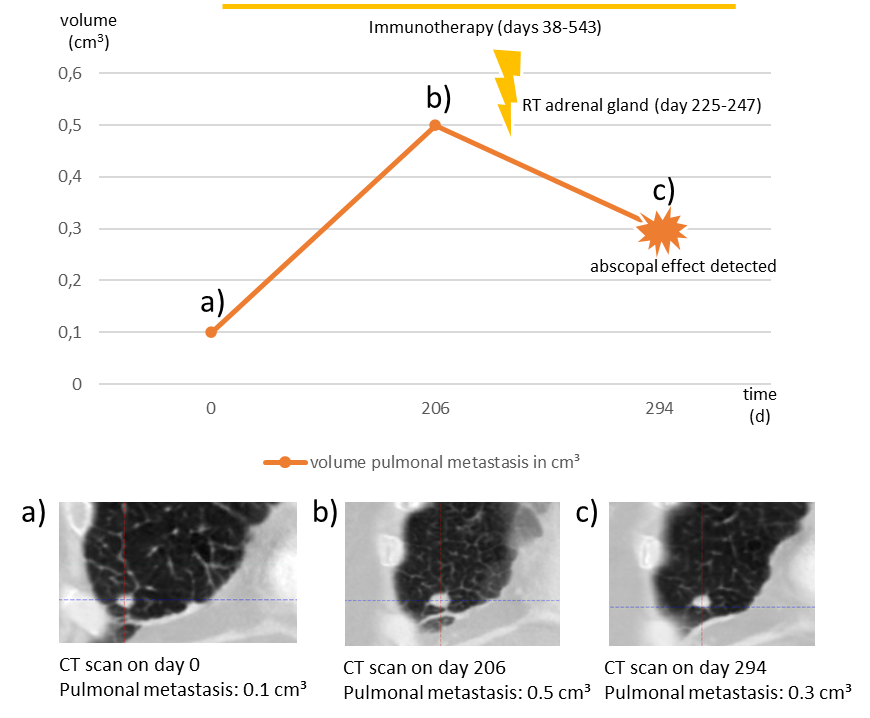
Suppl Case 2:

Patient 7 (Suppl Fig. 2) underwent IO for 505 days and underwent radiation therapy of the right adrenal gland from day 225 to 247 after the baseline CT for IO. The AbE was observed in a pulmonary metastasis on day 294 after the baseline CT for IO. The volume of the pulmonary metastasis changed as follows: initially 0.1 cm³ on day 0, increasing to 0.5 cm³ by day 206, and then reducing to 0.3 cm³ by day 294. This reduction from 0.5 cm³ to 0.3 cm³ was a 40% decrease. The AbE was shown 88 days after the completion of radiation therapy. The corresponding graphical evidence is shown below.

Suppl Fig. 2 - Patient 7

Suppl Case 3:


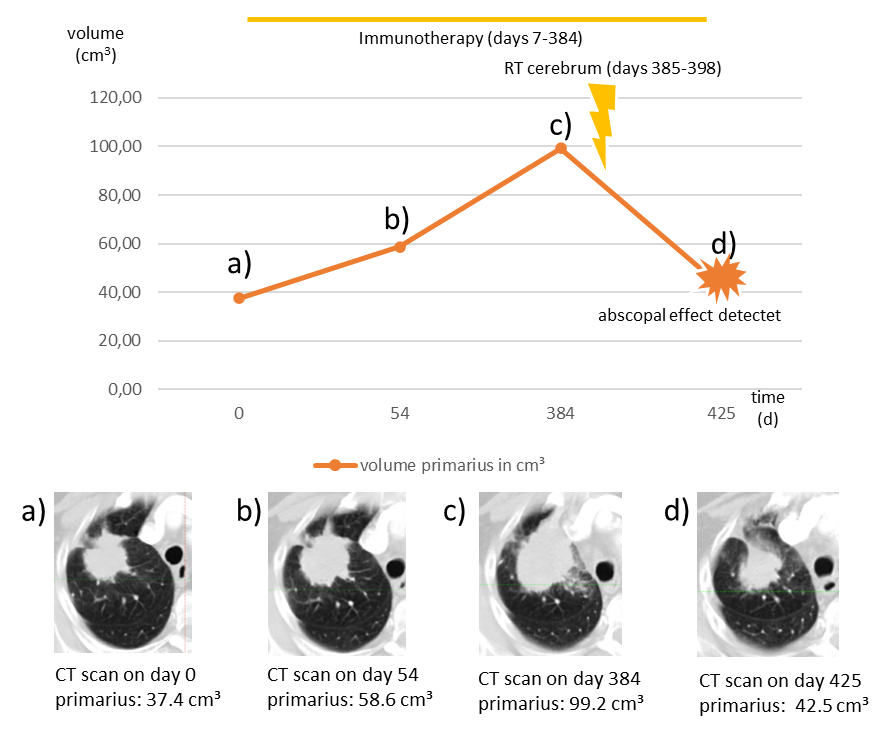
Patient 8 (Suppl Fig. 3) received IO for 377 days and underwent radiation therapy to the cerebrum from day 385 to day 398 after the baseline CT for IO. The primary tumor served as the abscopal site. Initially, the tumor volume was 37.4 cm³ on day 0, which increased to 58.6 cm³ by day 54 and further to 99.2 cm³ by day 384. Following this, the volume significantly decreased to 42.5 cm³ by day 425. This reduction from 99.2 cm³ to 42.5 cm³ was a 57.2% decrease. The AbE was shown 41 days after the completion of radiation therapy. The corresponding graphical evidence is shown beside.

Suppl Fig. 3 - Patient 8


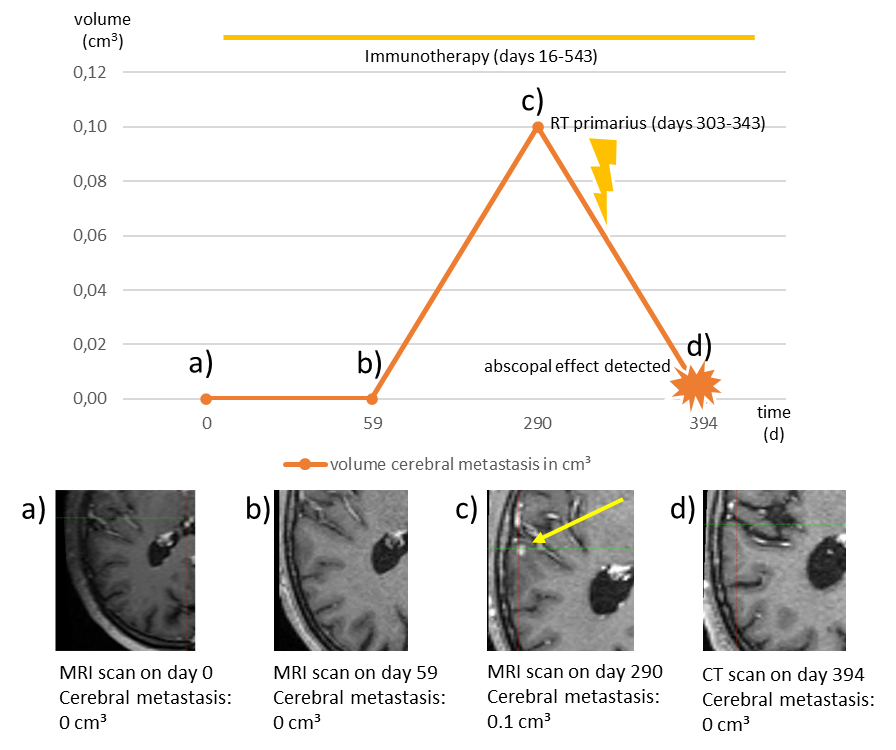
Suppl Case 4:

Patient 9's (Suppl Fig. 4) IO spanned approximately 526 days, with radiation therapy directed at the primary tumor from day 303 to day 343 after the baseline CT for IO. The AbE was observed in a cerebral metastasis. Initially, the metastasis was absent (0 cm³) on day 0 and day 59, appearing as 0.1 cm³ by day 290, and then disappearing again by day 394. The AbE was shown 104 days after the completion of radiation therapy. The corresponding graphical evidence is shown below.

Suppl Fig. 4 - Patient 9


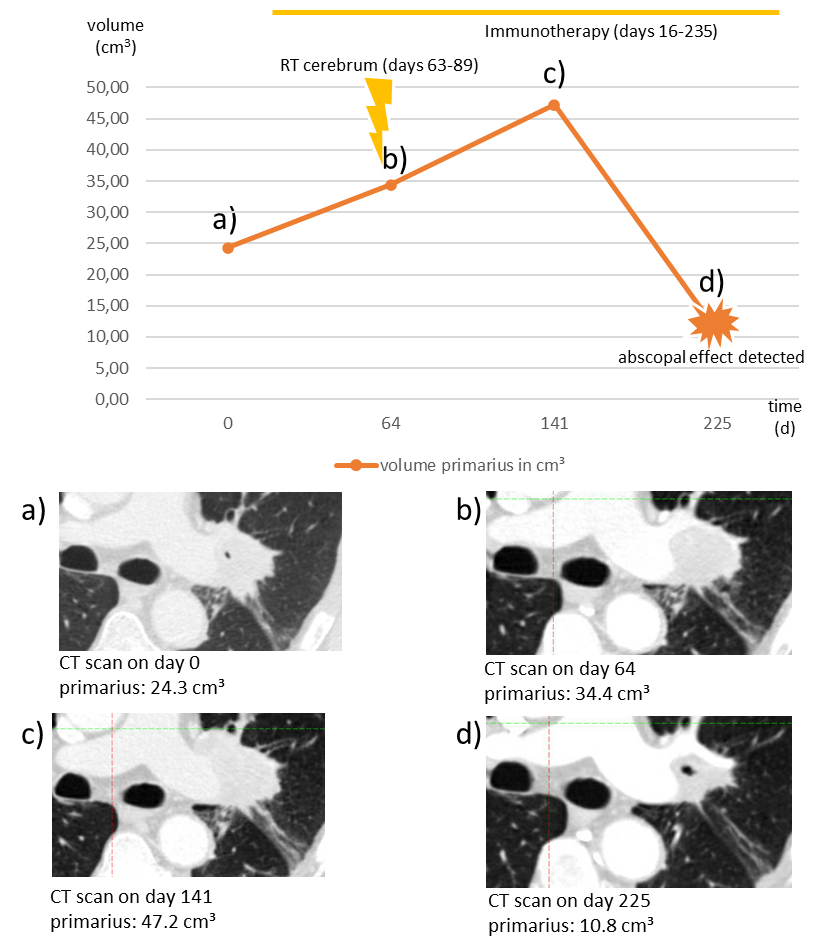
Suppl Case 5:

Patient 16 (Suppl Fig. 5) underwent IO for approximately 219 days, with radiation therapy to the cerebrum from day 63 to day 89 after the baseline CT for IO. The primary tumor was the site of the AbE. The volume of the tumor initially increased from 24.3 cm³ on day 0 to 34.4 cm³ by day 64 and 47.2 cm³ by day 141, before significantly reducing to 10.8 cm³ by day 225. This reduction from 47.2 cm³ to 10.8 cm³ was a 77.1% decrease. The AbE was shown 84 days after the completion of radiation therapy. The corresponding graphical evidence is shown below.

Suppl Fig. 5 - Patient 16


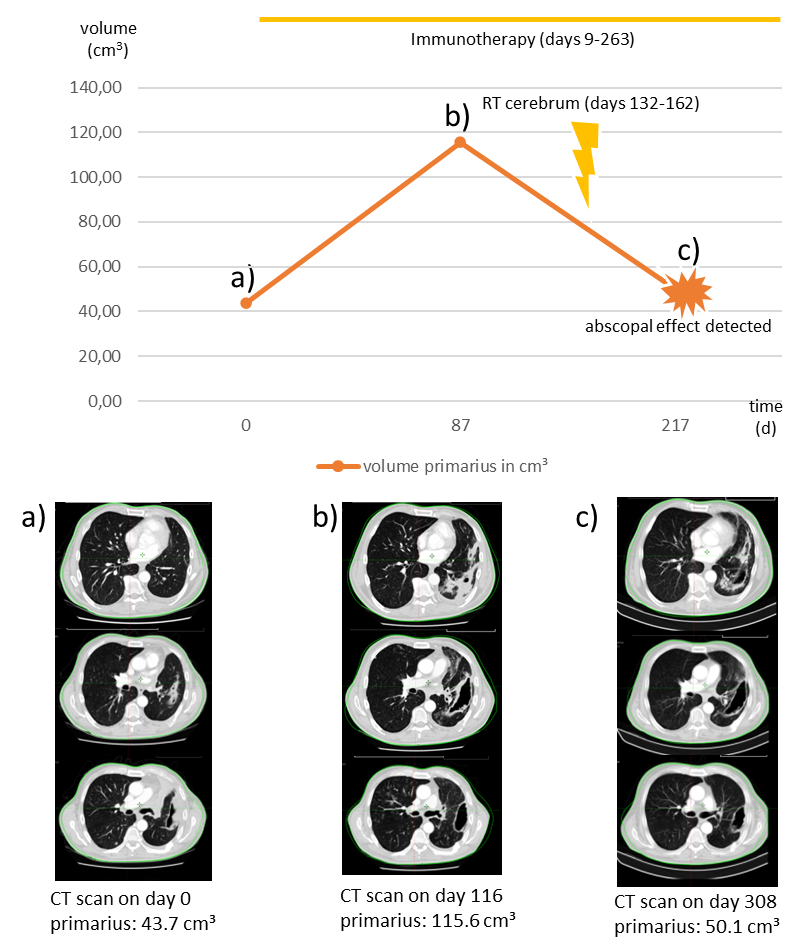
Suppl Case 6:

Patient 14 (Suppl Fig. 6) underwent IO for approximately 255 days, with radiation therapy targeting a cerebral metastasis from day 132 to day 162 after the baseline CT for IO. The primary tumor exhibited the AbE. The volume of the tumor was initially 43.7 cm³ on day 0, increased to 115.6 cm³ by day 116, and then significantly reduced to 50.1 cm³ by day 217. This reduction from 115.6 cm³ to 50.1 cm³ was a 56.7% decrease. The AbE was shown 101 days after the completion of radiation therapy. The corresponding graphical evidence is shown below.

Suppl Fig. 6 - Patient 14
